# Gamification With a Support Partner and Postoperative Mobility in Older Adults Undergoing Radical Cystectomy: The MOVE UP Randomized Clinical Trial

**DOI:** 10.1001/jamanetworkopen.2024.53037

**Published:** 2025-01-13

**Authors:** Daniel Lee, Charles Rareshide, Angira Mondal, Mitesh S. Patel, S. Ryan Greysen

**Affiliations:** 1 Division of Urology, Perelman School of Medicine, University of Pennsylvania, Philadelphia; 2 Corporal Michael Crescenz Veterans Affairs Medical Center, Philadelphia, Pennsylvania; 3 Center for Healthcare Incentives and Behavioral Economics, University of Pennsylvania, Philadelphia; 4 Leonard Davis Institute, University of Pennsylvania, Philadelphia; 5 Ascension Health, St Louis, Missouri; 6 Division of Hospital Medicine, Perelman School of Medicine, University of Pennsylvania, Philadelphia

## Abstract

This randomized clinical trial examines the effects of a gamified program for older adults to meet daily and weekly step goals following radical cystectomy procedures.

## Introduction

Early ambulation is a core tenet of recovery after major surgery to prevent postoperative complications. Patients who maintain their mobility postoperatively have lower complication rates, shorter hospital stays, and fewer readmissions.^1,2,3^ Although wearable activity monitors have been used to measure postoperative mobility,^1,2^ few studies have tested interventions to improve mobility.^4,5^ This randomized clinical trial sought to measure the effects of a gamified text message–based program to help older adults meet mobility goals following radical cystectomy surgery.

## Methods

The Mobility and Outcomes for Validated Evidence for Urology Patients (MOVE-UP) randomized clinical trial enrolled participants undergoing surgery at the Hospital of the University of Pennsylvania between November 2019 and September 2021 (protocol in Supplement 1). All participants set daily step goals and wore an activity tracking device (FitBit Inc) for presurgery baseline through 12 weeks after discharge. Following surgery, intervention patients played a mobility game with points for achieving daily goals and levels for weekly goals (eAppendix 1 in Supplement 2).

The study protocol was approved by the institutional review board for the University of Pennsylvania. Each patient provided written informed consent. This trial was registered with ClinicalTrials.gov (NCT04314778), and followed Consolidated Standards of Reporting Trials (CONSORT) reporting guidelines (eFigure in Supplement 2). All investigators and data analysts were masked to group assignments until analysis was completed.

Our primary outcome was in-hospital change in mean daily steps after surgery and before discharge. Secondary outcomes were proportion of discharges to postacute care facilities, posthospital change in steps from week 1 to 12 weeks after discharge, and rehospitalizations after discharge. Significance for all analyses was 2-sided *P* < .05. Further details on analysis are available in eAppendix 2 of Supplement 2.

## Results

A total of 61 participants were randomized with no demographic differences between groups (mean [SD] age, 66.2 (11.3) years; 46 male [75.4%]; 8 Black [13%], 50 White [82%], 3 other [5%]) (Table). Participants took a mean (SD) preoperative baseline 5385 (3536) steps, and median (IQR) length of stay was 7 (5-11) days. Forty-six participants (75%) received open approach procedures, and 26 participants (43%) received neoadjuvant chemotherapy. No adverse events were reported.

**Table.** Sociodemographics and Clinical Characteristics

Characteristic	Participants, No. (%)		
	Control (n = 31)	Intervention (n = 30)	Total (N = 61)
**Sociodemographics**			
Age, mean (SD), y	67 (12.4)	65.3 (10.2)	66.2 (11.3)
Sex			
Female	6 (19)	9 (30)	15 (25)
Male	25 (81)	21 (70)	46 (75)
Race and ethnicity			
White non-Hispanic	25 (81)	25 (83)	50 (82)
Black non-Hispanic	4 (13)	4 (13)	8 (13)
Other^a^	2 (7)	1 (3)	3 (5)
Marital status			
Single	3 (10)	6 (20)	9 (15)
Married	21 (68)	20 (67)	41 (67)
Other	7 (23)	4 (13)	11 (18)
Household income, mean (SD), $	81 623 (29 783)	91 706 (35 095)	86 582 (32 629)
**Clinical characteristics**			
BMI, mean (SD)	27.2 (4.4)	29.6 (6.3)	28.4 (5.5)
Current smoker	3 (10)	2 (7)	5 (8)
Diabetes	5 (16)	4 (13)	9 (15)
Myocardial infarction	1 (3)	1 (3)	2 (3)
Heart failure	0	0	0
OSA	1 (3)	1 (3)	2 (3)
COPD	1 (3)	1 (3)	2 (3)
Stroke	1 (3)	0	1 (2)
Length of stay, mean (IQR), d	6.5 (5-11)	7 (5-11)	7 (5-11)
Neoadjuvant chemotherapy	14 (45)	12 (40)	26 (43)
Robotic surgical approach	8 (26)	7 (23)	15 (25)
Preoperative baseline steps, mean (SD), No.	5411 (2481)	5360 (4369)	5385 (3536)

Abbreviations: BMI, body mass index (calculated as weight in kilograms divided by height in meters squared); COPD, chronic obstructive pulmonary disease; OSA, obstructive sleep apnea.

^a^ Other included Asian and American Indian.

During hospitalization, 23 participants (15 control, 8 intervention) wore the activity tracking device for 2 or more days of step data before discharge; intervention patients had a nonsignificant increase in steps compared with control (adjusted difference, 689 steps; 95% CI, −541 to 1919 steps; *P* = .27) (Figure). Intervention participants had fewer discharges to a skilled nursing facility or subacute rehabilitation (0 of 8 participants) compared with control (5 of 15 participants).

**Figure.**
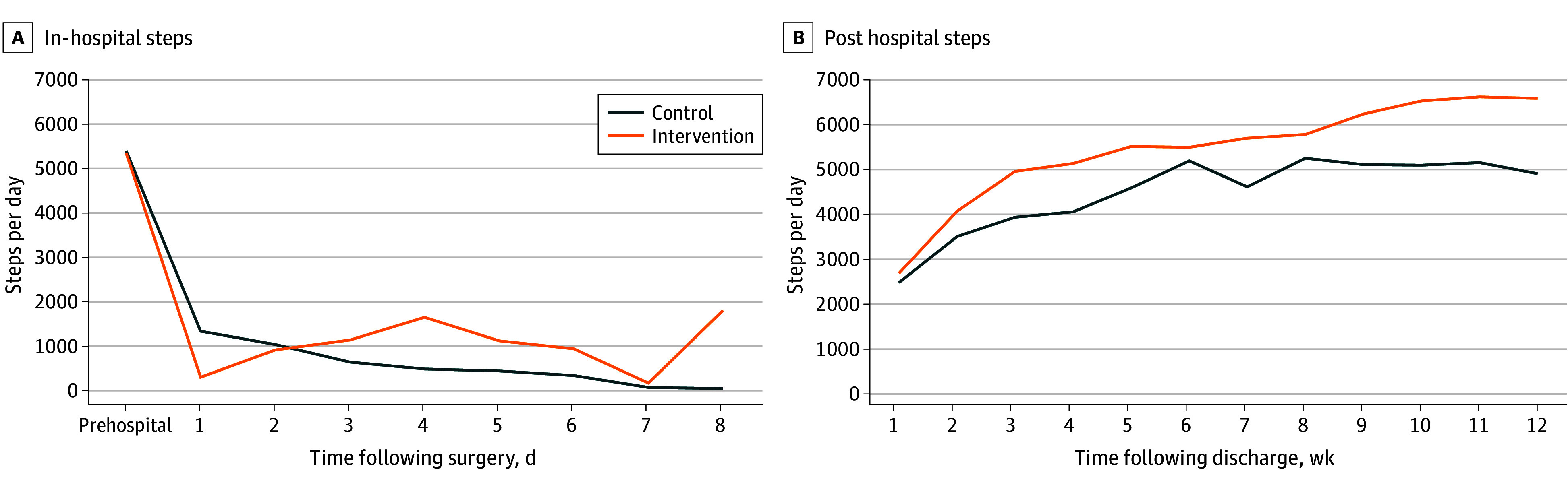
Prehospital, In-Hospital, and Posthospital Steps for All Participants With 2 or More Days of Data in Each Period

After discharge, 53 participants (28 control, 25 intervention) completed the 12-week postdischarge period; intervention patients had a nonsignificant increase in steps compared with control (adjusted difference 793 steps; 95% CI −446 to 2031 steps; *P* = .21) (Figure). Intervention participants had fewer readmissions at 90 days (5 of 30 [17%] vs 11 of 31 [35%]), a difference that was not statistically significant (*P* = .09). Intervention participants also returned to their preoperative baseline by week 5 postdischarge and continued to increase above baseline after week 7, whereas control patients did not return to baseline by 12 weeks (Figure).

## Discussion

This pilot study of a text-based mobility game played with a support partner demonstrated feasibility but did not significantly increase steps per day from baseline. Importantly, our results showed intervention participants returned to their preoperative baseline by week 5 postdischarge and continued to increase, whereas control patients had not returned to baseline by 12 weeks. Although we did not prespecify subgroup analyses, these results were particularly obvious for participants who experienced a larger decline from preoperative baseline steps. Future studies may wish to focus on time for return to preoperative baseline as the primary objective and consider targeting individuals with larger postoperative decline from preoperative baseline.

Limitations include limited bedside study support during the in-hospital phase and small sample size. Greater bedside support could increase in-hospital adherence to protocol (wearing the device sooner after surgery) to assess the impact of the intervention before discharge. A larger sample would provide more power to assess significance of trends in postacute outcomes we observed (fewer discharges to postacute facilities and readmissions). Strengths include fully automated delivery of the intervention via text messages which was acceptable in this older population and feasibility of fully remote monitoring of steps after discharge.

## Notes

Supplement 1.
Trial Protocol

Supplement 2. **eAppendix.** Study Methods **eFigure.** CONSORT Flow Diagram

Supplement 3.
Data Sharing Statement
