# Supplementary material for: Gamification With a Support Partner and Postoperative Mobility in Older Adults Undergoing Radical Cystectomy: The MOVE UP Randomized Clinical Trial
Source: JAMA Netw Open. 2025 Jan 13;8(1):e2453037. doi: 10.1001/jamanetworkopen.2024.53037 (PMC11731219; doi:10.1001/jamanetworkopen.2024.53037)
Supplement: Supplement 1. — Trial Protocol [file jamanetwopen-e2453037-s001.pdf]

Using patient centered data and behavioral economics to improve mobility and reduce readmissions after radical cystectomy

Study Protocol

Outline

1. Abstract
2. Overall objectives
3. Aims
    3.1 Primary outcome
    3.2 Secondary outcomes
4. Background
5. Study design
    5.1 Design
    5.2 Study duration
    5.3 Target population
    5.4 Accrual
    5.5 Key inclusion criteria
    5.6 Key exclusion criteria
6. Subject recruitment
    6.1 General
    6.2 Populations vulnerable to undue influence or coercion
7. Subject compensation
8. Study procedures
    8.1 Consent
    8.2 Procedures
9. Analysis plan
10. Investigators
11. Human research protection
    11.1 Data confidentiality
    11.2 Subject confidentiality
    11.3 Subject privacy
    11.4 Data disclosure
    11.5 Safety monitoring
    11.6 Risk/benefit
        11.6.1 Potential study risks
        11.6.2 Potential study benefits
        11.6.3 Risk/benefit assessment
        11.6.4 Alternatives to participation

1. Abstract

Major surgical procedures for cancer treatment are complex and are very difficult to recover from. The majority of patients have a complication, and may have long-term cognitive or physical problems. Finding ways to improve mobility and walking may help improve how patients recover from surgery. Only 1 in 4 hospitalized patients walk outside their room during hospitalization, and patients spend 95% of their hospitalized time in bed. However, there are few studies to show if this can be successful, and how to best motivate patients to walk. We want to evaluate how patients do after a radical cystectomy (removal of the bladder), to see if those receiving social support walk more and have less complications than those who receive the usual care. Our central hypothesis is that social incentives and support will increase how patients walk after complex surgeries, and will decrease healthcare utilization such as readmissions. This project will be a randomized trial comparing standard practice compared to social incentives and gamification postoperatively. The primary outcome is the number of steps per day, with secondary outcomes of postoperative readmissions. This will be the first trial of its kind to test if a simple strategy to increase walking in patients with bladder cancer can improve how patients do after surgery, and may be scalable for other major surgeries.

2. Overall objectives

The primary objective is to assess the effectiveness of a social incentive-based gamification intervention after radical cystectomy to increase physical activity postoperatively. We will explore patients' physical activity postoperatively at the time of discharge and if receiving the social intervention affects the amount of physical activity. A secondary objective will explore the effect of postoperative social incentives on improving physical activity at 3 months post-discharge. We will also explore changes in patient functional decline, cognitive function, postoperative delirium, SNF placement, readmission rates, and postoperative complication rates graded by the Clavien-Dindo classification.

3. Aims

3.1 Primary outcome

The primary outcome variable is the change in mean daily step count from the baseline period after a radical cystectomy to the time of hospital discharge postoperatively.

3.2 Secondary outcome

The secondary outcome variable is the change in mean daily step count from the postoperative baseline to 3 months after discharge from the hospital.

3.3 Exploratory outcome

The exploratory outcome variable is a composite of placement to skilled nursing facility, 30-day hospital readmission, postoperative complication rates, cognitive function, postoperative delirium rates, and functional decline postoperatively.

4. Background

Treatment for bladder cancer is complex and disproportionately affects a vulnerable elderly population. The median age at diagnosis for bladder cancer is 73 years,[1] and 30-60% of patients experiencing a postoperative complication with up to 30% readmitted within 90 days.[2-8] Preliminary evidence suggests that mobility is a potential lever to improve postoperative complications and readmissions.[9-11] However, only 1 in 4 hospitalized patients ever walk outside their room during hospitalization,[12,13] and previously ambulatory patients spend 95% of their hospitalized time in bed.[14,15] Despite a growing body of literature supporting the use of wearable technology to measure postoperative mobility,[16-18] few studies evaluate how best to implement mobility interventions for elderly patients undergoing surgery. There is no data to show how much immobility is dangerous, and how much mobility is needed to prevent adverse outcomes after major surgery.

Study design

5.1 Design

We will conduct a two-arm randomized, controlled trial during the postoperative period after a radical cystectomy for bladder cancer, comparing a control group that uses a wearable device to track physical activity to an intervention group that uses the same wearable devices and receives a supportive social incentive-based gamification intervention to adhere to a step goal program. Patients will be enrolled after being diagnosed with bladder cancer and scheduled to undergo a radical cystectomy. The median length of stay after a radical cystectomy is 8 days, with 90% being discharged within two weeks. Postoperatively, the baseline step count will be averaged by taking the steps taken during the first two days outside of the intensive care unit. There will be two postoperative phases: Phase I will be while the patient is hospitalized postoperatively. Phase II will be when the patient is discharged for three months.

5.2 Study duration

The study will begin in Summer 2019. The primary intervention period is from the time of diagnosis through the cystectomy and postoperative recovery to 3-months post-hospital discharge. We expect the entire study to take approximately 18 months to conduct and analyze outcomes.

5.3 Target population

Adults age 18 years or older who have bladder cancer and are undergoing a radical cystectomy

5.4 Accrual

We will aim to enroll and randomize 30 patients. We estimate that a sample size of at least 30 participants (15 per arm) will provide 80% power to detect a difference of 500 steps in the change in mean daily step count from baseline to discharge between intervention and control, using a 2-sided α of 0.05, assuming a baseline mean preoperative step count of 4000 steps and mean postoperative step count of 1074 steps (SD 955 steps), and accounting for a 10% dropout rate.

5.5 Key inclusion criteria

1) Age 18 years or older; 2) diagnosis of bladder cancer undergoing a radical cystectomy

5.6 Key exclusion criteria

1) Inability to provide informed consent; 2) does not have daily access to a smartphone compatible with the wearable device and not willing to use a device that we can provide them; 3) any other medical conditions that would prohibit participation in a physical activity program

6. Subject recruitment

6.1 General

The study team will use the electronic health record to identify potentially eligible patients in the Urology Department.

6.2 Populations vulnerable to undue influence or coercion

Not applicable

7. Subject compensation

Participants will receive $25 for enrolling and $25 for completing the discharge period, and $50 for completing the 3-month post-discharge period. Compensation will be provided in a form of Greenphire ClinCard.

8. Study procedures

8.1 Consent

Potential subjects will first have the study fully explained to them and all questions will be answered, whereupon a written informed consent will be obtained. All aspects of the protocol and all procedures will be disclosed to the subject during this informed consent. Each subject will be told that he/she is free to discontinue participation in the study at any time and that the investigators also reserve the right to discontinue the study for medical or other reasons. Participants will be provided with the paper consent form for signature and will be asked to either provide consent or decline participation in the study. In the paper copy of the consent, details regarding how to contact the research team via email or phone will be provided in the event a participant wants to withdraw from the study.

All patients who consent to this study will be debriefed in the same encounter during which they were approached.

- Clinical research coordinator will describe the study's use of deception and purpose.
- Patients will also be informed of reimbursement (total $100)

8.2 Procedures

Each week, the research coordinator will use an electronic health record query to generate a list of patients that are diagnosed with bladder cancer and scheduled for a radical cystectomy. At enrollment, the research coordinator will administer two additional screening questions to determine eligibility (owns a smart phone and able to provide consent) via the RedCap portal. If the participant successfully passes the screening questions, the patient can be consented to the study. The research coordinator will next present a series of surveys to the patient to be completed through the Way to Health portal. These validated instruments include: the Pittsburgh Sleep Quality Index, Karolinska Scale, Activity of Daily Living-Instrumental Activities of Daily Living Survey, Life Space Activity Survey, Mini-Nutritional Assessment Survey, Edmonton Symptom Assessment Survey, Treatment Burden Questionnaire, Lubbens Social Network Scale, Self Rated Health Survey, and Dementia Tool.

After completing the enrollment assessment, the research coordinator will show patients how to use the wearable device which can be worn on wrist (like a watch), carried in any pocket, or clipped to hospital gown.

Upon enrollment after diagnosis of bladder cancer and scheduled for cystectomy, participants will use the watch for one week to ensure that the data is captured accurately. After the radical cystectomy, there will be a two-day run-in period to estimate postoperative baseline step counts for all patients. For all patients with a baseline step count, they will be randomly assigned to control or intervention using block sizes of two. After discharge, all participants will be notified to complete post-discharge baseline surveys.

Participants in the control group will continue to have data collected passively. Participants in the control arm will only receive study communications when it is time for them to complete surveys in the Way to Health system.

Participants in the intervention arm will have a postoperative daily step goal that increases from baseline by 500 steps each day. At the beginning of each week, starting from the first day after the postoperative baseline is set (i.e. postoperative day 3), the participant receives 70 points (10 points for each day that week). If the participant does not meet their daily step goal, they lose 10 points from their balance. This leverages loss aversion, which has been demonstrated to motivate behavior change more effectively with losses than gains. At the end of each week if the participant has at least 40 points, they will move up a level (levels from lowest to highest: blue, bronze, silver, gold, platinum). If not, the participant will drop a level. All participants begin at the silver level. Each week, participants get a fresh set of 70 points. Participants will receive daily feedback for the step counts, and weekly feedback for their levels. Participants in the intervention arm will be asked to identify a family member or friend to be their support sponsor. A weekly report will be sent to this person with the participant's performance (points and level).

After discharge, the goals will be changed from daily step goals to weekly step goals to increase the weekly step counts by 10% each week to a maximum of 10,000 steps.

Participants in both arms will also receive a notification when it is time to complete surveys in the Way to Health system (5, 9, and 13 weeks after hospital discharge). 3 days after the surveys are sent to participants, a message will be sent reminding them to complete the surveys if they have not already done so. Upon completion of the final set of surveys, $50 will be processed for participants in both arms.

9. Analysis plan

In our primary analyses, we will use multiple imputation for missing data and use linear mixed effects models to compare the change in mean daily step count from baseline to hospital discharge (ie postoperative Phase I time period), adjusting for baseline step count and time. To test of the robustness of our findings we will also evaluate models using collected data without imputation. All hypothesis tests will be two-sided using a two-sided alpha of 0.05 as our threshold for statistical significance. In secondary analyses, a similar linear mixed effects model

will compare the change in mean daily step counts from postoperative baseline to 3 months post-discharge (Phase II time period).

In exploratory analyses, we will evaluate how differences in postoperative step counts are associated with outcome measures. We will also conduct a qualitative analysis to evaluate participant perceptions of the intervention.

10. Investigators

Daniel Lee, MS, MS is the Co-Principle Investigator. He is an Assistant Professor of Urology and Surgery. He has extensive experience in leveraging health informatics tools to promote clinical decision making and healthy behaviors for patients. He currently spends 70% of his effort on clinical and teaching activities and 30% on research.

Ryan Greysen, MD, MHS, MA is the Co-Principle Investigator. He is Chief of the Section of Hospital Medicine in the Division of General Internal Medicine and an Assistant Professor of Medicine at the Perelman School of Medicine at the University of Pennsylvania. He has past experience leading studies of older, hospitalized adults focused on functional vulnerability and outcomes including hospital readmission. He currently spends 75% of his effort on research and 25% on clinical and administrative activities.

Thomas Guzzo, MD, MPH is the Co-Investigator. He is the Chairman of the Division of Urology and has led several clinical trials, and is currently the site-PI for a large multi-institutional trial investigating a novel therapeutic agent for patients with BCG refractory non-muscle invasive bladder cancer. He has extensive clinical expertise in bladder cancer, and is a world renowned expert in muscle invasive bladder cancer management, role of cystectomies for bladder cancer, and methods to improve outcomes. He currently spends 80% of his effort in clinical and teaching activities, and 20% on research and administrative work.

11. Human research protection

11.1 Data confidentiality

Paper-based records will be kept in a secure location and only be accessible to personnel involved in the study. Computer-based files will only be made available to personnel involved in the study through the use of access privileges and passwords. Wherever feasible, identifiers will be removed from study-related information. Precautions are in place to ensure the data are secure by using passwords and encryption, because the research involves web-based surveys.

11.2 Subject confidentiality

Research material will be obtained from participant surveys, from the wearable devices, and from the electronic health record. All participants will provide informed consent for access to these materials. The data to be collected include demographic data (e.g., age, sex, self-identified race), outcome data, and daily activity data collected by the wearable device. Research material that is obtained will be used for research purposes only. The same procedure used for the analysis of automated data sources to ensure protection of patient information will be used for the survey data, in that patient identifiers will be used only for linkage purposes or to contact patients. The study identification number, and not other identifying information, will be used on all data collection instruments. All study staff will be reminded to appreciate the confidential

nature of the data collected and contained in these databases. The Penn Medicine Academic Computing Services (PMACS) will be the hub for the hardware and database infrastructure that will support the project and is where the Way to Health web portal is based. The PMACS is a joint effort of the University of Pennsylvania's Abramson Cancer Center, the Cardiovascular Institute, the Department of Pathology, and the Leonard Davis Institute. The PMACS provides a secure computing environment for a large volume of highly sensitive data, including clinical, genetic, socioeconomic, and financial information. Among the IT projects currently managed by PMACS are: (1) the capture and organization of complex, longitudinal clinical data via web and clinical applications portals from cancer patients enrolled in clinical trials; (2) the integration of genetic array databases and clinical data obtained from patients with cardiovascular disease; (3) computational biology and cytometry database management and analyses; (4) economic and health policy research using Medicare claims from over 40 million Medicare beneficiaries. PMACS requires all users of data or applications on PMACS servers to complete a PMACS-hosted cybersecurity awareness course annually, which stresses federal data security policies under data use agreements with the university. The curriculum includes Health Insurance Portability and Accountability Act (HIPAA) training and covers secure data transfer, passwords, computer security habits and knowledge of what constitutes misuse or inappropriate use of the server. We will implement multiple, redundant protective measures to guarantee the privacy and security of the participant data. All investigators and research staff with direct access to the identifiable data will be required to undergo annual responsible conduct of research, cybersecurity, and HIPAA certification in accordance with University of Pennsylvania regulations.

Data will be stored, managed, and analyzed on a secure, encrypted server behind the University of Pennsylvania Health System (UPHS) firewall. This server was created for projects conducted by the Penn Medicine Nudge Unit related to physician and patient behavior at UPHS. All study personnel that will use this data are listed on the IRB application and have completed training in HIPAA standards and the CITI human subjects research. Data access will be password protected. Whenever possible, data will be deidentified for analysis.

11.3 Subject privacy

Enrollment will include a description of the voluntary nature of participation, the study procedures, risks and potential benefits in detail. The enrollment procedure will provide the opportunity for potential participants to ask questions and review the consent form information with family or caregivers prior to making a decision to participate. Participants will be told that they do not have to answer any questions if they do not wish and can drop out of the study at any time, without affecting their medical care or the cost of their care. They will be told that they may or may not benefit directly from the study and that all information will be kept strictly confidential, except as required by law. Subjects will be given a copy of the consent document. All efforts will be made by study staff to ensure subject privacy.

11.4 Data disclosure

The following entities, besides the members of the research team, may receive protected health information (PHI) for this research study: -Greephire, the company which processes study-related payments. Patient addresses and account balances will be stored on their secure computers. -Fitbit, Inc., the company that designs and manufactures the wearable devices used in

the study to track participant physical activity. -Twilio, Inc., the company which processes some study-related messages. Twilio will store patients' phone numbers on their secure computers. -Qualtrics, Inc., the company which processes most study-related surveys. Qualtrics will house de-identified answers to these surveys on their secure servers. -The Office of Human Research Protections at the University of Pennsylvania. -Federal and state agencies (for example, the Department of Health and Human Services, the National Institutes of Health, and/or the Office for Human Research Protections), or other domestic or foreign government bodies if required by law and/or necessary for oversight purposes.

11.5 Safety monitoring

At the time of enrollment in the hospital, all patients will be given anticipatory guidance on when to seek medical attention (e.g. when to call their nurse or doctor should they feel dizzy, short of breath, chest pain, lightheaded, unstable, or otherwise unwell while ambulating). In addition, participants will be asked to report to the study team any episodes of these symptoms that occur during ambulation during visits with research staff (the research assistant will check in periodically with enrolled patients to ensure devices are working properly and troubleshoot any issues encountered by patients/caregivers or nursing). Patients/caregivers will also be reminded that they can always contact the study team by phone or email at any time (contact information will be given at the beginning of the study and will also be posted on the Way to Health platform, which can be accessed at any time by the participant). If any concerns of a participant event are identified, the study coordinator will reach out to the participant and complete the event reporting form. This form will be reviewed with the study PI to determine if any action is needed and if the participant can continue safely in the study. Any identified adverse events will be reported to the Institutional Review Board. For patients on floors where the mobility protocol is in place, mobility goals are revised daily by the bedside nurse caring for the patient. If patient is noted to be weaker, the nurse will automatically reduce the mobility goal for the day per the mobility protocol (this happens without regard to research protocol).

11.6 Risk/benefit

11.6.1 Potential study risks

The major potential risk of this study is a breach of participant confidentiality. We will minimize this risk of confidentiality breach by linking individual identifying information with participant ID numbers only in one single secure file that will only be accessed by the study team in the case of an adverse medical event, participant dropout, or if otherwise deemed necessary by the Principal Investigator. All other identifying information will be discarded after initial contact with the Study Coordinator. All other members of the research team will be able to view only participant ID numbers. Additionally, participants will receive guidance from the research coordinator on when to seek medical attention (call for their nurse or doctor in the hospital) and a reporting protocol is in place to capture any changes in symptoms with physical activity.

11.6.2 Potential study benefits

Through participation in this study, each participant will have the potential to increase their physical activity, which could improve their health and reduce their risk for functional decline, falls, nursing home placement, or hospital readmission. If this approach is effective, it could have tremendous benefits for society if adopted on a wide scale to help individuals increase physical activity during hospitalization to avoid harms of complex surgery and hospitalization such as

functional decline or nursing home placement. It is expected that other people will gain knowledge from this study and that participation could help understand how to effectively motivate people to become more physically active. Participants may also receive no benefit from their participation in the study.

11.6.3 Risk/benefit assessment

Anticipated risks of this study should be minimal and the risk/benefit ratio is very favorable. To minimize the chance for serious and unexpected adverse events, study participants will be screened through exclusion criteria. Participants that increase physical activity may improve their health and reduce their risk for hospital-associated functional decline, falls, nursing home placement, or hospital readmission.

11.6.4 Alternatives to participation

Patients do not have to be enrolled in the study in order to participate in activities to promote healthy mobility and overall patient engagement - all patients (regardless of study status) will receive usual care in these areas including physical therapy, nursing assistance with ambulation as needed, etc. Participation in the study merely entails wearing sensors to quantify mobility and answering questions from enrollment and exit surveys. Patients may receive feedback on their mobility from healthcare providers (nurses, physical therapists, doctors) without agreeing to participate in the study and wear mobility sensors.
