# Supplementary material for: Gamification With a Support Partner and Postoperative Mobility in Older Adults Undergoing Radical Cystectomy: The MOVE UP Randomized Clinical Trial
Source: JAMA Netw Open. 2025 Jan 13;8(1):e2453037. doi: 10.1001/jamanetworkopen.2024.53037 (PMC11731219; doi:10.1001/jamanetworkopen.2024.53037)
Supplement: Supplement 2. — eAppendix. Study Methods eFigure. CONSORT Flow Diagram [file jamanetwopen-e2453037-s002.pdf]

Supplemental Online Content

Lee D, Rareshide C, Mondal A, Patel MS, Greysen SR. Effect of gamification with a support partner to increase postoperative mobility in older adults undergoing radical cystectomy. *JAMA Netw Open.* 2025;8(1):e2453037. doi:10.1001/jamanetworkopen.2024.53037

eAppendix. Study Methods

eFigure. CONSORT Flow Diagram

This supplemental material has been provided by the authors to give readers additional information about their work.

© 2025 Lee D et al. *JAMA Network Open*.

eAppendix. Study Methods

Gamification is based on behavior change science to motivate behavior change
Gamification, the approach of applying game elements such as levels and points in non-game contexts, is based on the science of behavior change. Principles derived from psychology and economics help explain mechanisms of short-term decisions (whether or not to take a walk on a given day) to better align actions with long-term goals (such as recovery from cancer surgery). One example is the use of a game to deliver immediate feedback rather than delayed feedback on achieving an individual's activity goal. The rules of the game (see below) can be used to deliver daily feedback on steps each day rather than giving feedback on a weekly or less frequent basis. This can be paired with another behavioral science concept known as loss aversion which asserts that participants are more motivated by a game that causes them to lose points if they miss their daily goal rather than being rewarded by gaining points if the goal is met. Leveraging these decision mechanisms within a simple game can make behavior change easier to initiate and sustain.

Gamification intervention components:

Run-in period to establish baseline, pre-operative activity: After enrolling in the trial during an in-person clinic visit which is 4-6 weeks before surgery, all participants are instructed to spend two weeks getting accustomed to their FitBit device. During this run-in period, we determine the pre-operative baseline level of physical activity with an average daily step count using the second week of data. The first week of data is ignored to diminish the potential upward bias from higher activity during initial use (e.g. shiny new toy effect). To prevent potential downward bias, we omitted any total daily value less than 100 steps because prior studies suggest these values are unlikely to represent meaningful activity.

Randomization and goals: Participants were randomized 1:1 to gamification vs. a control group. Both groups are given a goal of 10% increase in mean daily steps after discharge that is personalized according to their baseline. Control patients receive no feedback on whether they achieve their daily step goal although they can see their steps on the FitBit device and in the FitBit app.

Gamification: Only intervention patients receive the game which is delivered via daily text messages. The game runs automatically and does not require any effort on the part of the participant to 'play' the game other than to strive for physical activity goals. The components of the gamification intervention are listed below and described in **Table 1**. Although gamification leverages multiple behavioral mechanisms, these are invisible to participants.

**Table 1**<br>**Gamification component**	**Application to intervention design**
Pre-commitment [1, 2]	Each individual signs a pledge committing to do their best to adhere to the game for the duration of the study
Personalized goals [3, 4]	Participants complete a 2-week run-in to establish baseline for goal increase
Daily feedback [5, 6]	Participants receive daily feedback on meeting their step goal and weekly feedback on game levels
Loss aversion [7, 8]	Points are deducted each day that participant does not meet daily step goal
Goal gradients [9, 10]	Participants move up or down game levels based on weekly performance
Temporal landmarks [11, 12]	Each Monday participants are endowed with points for that week for a fresh start

<u>Pre-commitment</u>: After randomization but before the game begins, each participant signs a pre-commitment pledge agreeing to try his or her best to achieve the daily step goal. Pre-commitment has been demonstrated to help motivate behavior change [1, 2]. The pledge is signed electronically on the Way to Health (W2H) platform. Each time a participant signs into W2H, the pledge will be displayed along his or her digital signature to remind them of the commitment. The W2H platform also automatically pulls data from the FitBit device and sends text messages to participants about whether they achieved their daily step goal as well as game points retained or last as described below.

© 2025 Lee D et al. *JAMA Network Open*.

Points: At the start of each week, each participant will receive 70 points (10 for each day that week). Points are endowed at the start of the week, rather than awarded after daily goal achievement, to leverage prospect theory [7] which has demonstrated that loss-framing is more effective at motivating behavior change than gain-framing [7, 8].

Each day, if a participant achieved their daily step goal on the prior day, he or she keeps his or her points. However, if the goal is not met, 10 points are deducted from their balance. Points are replenished at the start of the week to leverage the ‘fresh start effect’ the concept that individuals are more motivated for aspirational behavior around temporal landmarks such as the start of the week [11].

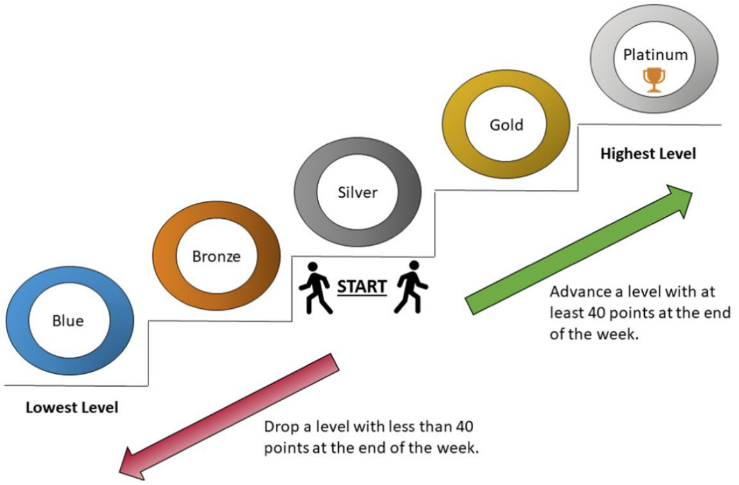

Figure 1. Gain and loss of points during gamification

Levels: At the end of the week, if the participant has at least 40 points then the he or she will advance up one level as shown in **Figure 1** [12]. The levels from lowest to highest are blue, bronze, silver, gold, and platinum. If the participant has less than 40 points he or she will move down a level. This creates a sense of achievable goals (goal gradients) and longer-term loss aversion [9, 10]. Instead of beginning in the bottom level, participants will start in the middle at silver so they will feel the loss of dropping to bronze if they do not keep enough points in the first week.

Participants can see their current level in the game by logging into their account on the Way to Health platform which they created at the beginning of the study. An example of this patient-facing dashboard is shown in **Figure 2**.

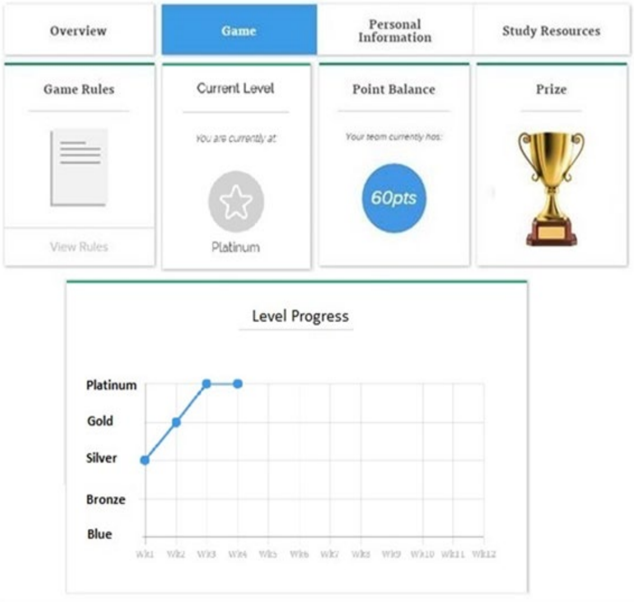

Figure 2: Gamification participant dashboard

Support Partner: We also leveraged social networks to increase participant accountability, sense of accomplishment, and overall engagement in this mobility game. Participants identified a family member or friend to be a support sponsor who was asked via email at the start of the study to provide support and encouragement to the participant to help them achieve their mobility goals. This partner received a weekly email update report on participant performance including points and level.

References

1. Rogers T, Milkman KL, Volpp KG. Commitment devices: using initiatives to change behavior. JAMA. 2014;311(20):2065-2066
2. Schwartz J, Mochon D, Wyper L, Maroba J, Patel D, Ariely D. Healthier by precommitment. Psychological science. 2014;25(2):118–546.
3. Fishbach, A. and R. Dhar (2005). "Goals as excuses or guides: The liberating effect of perceived goal progress on choice." Journal of Consumer Research 32(3): 370-377.
© 2025 Lee D et al. *JAMA Network Open*.

4. Bailey, R. R. (2019). "Goal Setting and Action Planning for Health Behavior Change." Am J Lifestyle Med 13(6): 615-618.
5. O'Donoghue, T. and M. Rabin (2000). "The economics of immediate gratification." Journal of Behavioral Decision Making **13**(2): 233-250.
6. Schroeder, J. and A. Fishbach (2015). "How to motivate yourself and others? Intended and unintended consequences." Research in Organizational Behavior **35**: 123-141.
7. Kahneman D, Tversky A. Prospect Theory: An Analysis of Decision under Risk. Econometrica. 1979;47(2):263.
8. Zeelenberg M, Pieters R. Consequences of regret aversion in real life: The case of the Dutch postcode lottery. Organizational Behavior and Human Decision Processes. 2004;93(2):155-168.
9. Kawachi, I. (2017). "It’s all in the game—The uses of gamification to motivate behavior change." JAMA Internal Medicine **177**(11): 1593-1594.
10. Huang SC, Zhang Y, Broniarczyk SM. So near and yet so far: the mental representation of goal progress. J Pers Soc Psychol. 2012 Aug;103(2):225-41.
11. Dai H, Milkman KL, Riis J. The Fresh Start Effect: Temporal Landmarks Motivate Aspirational Behavior. Management Science. 2014;60(10):2563-2582.
12. Waddell KJ, Patel MS, Clark K, Harrington TO, Greysen SR. Leveraging insights from behavioral economics to improve mobility for adults with stroke: Design and rationale of the BE Mobile clinical trial. Contemp Clin Trials. 2021 Aug;107:106483. doi: 10.1016/j.cct.2021.106483. Epub 2021 Jun 12. PMID: 34129953.

© 2025 Lee D et al. *JAMA Network Open*.

Supplement 2: CONSORT Flow Diagram

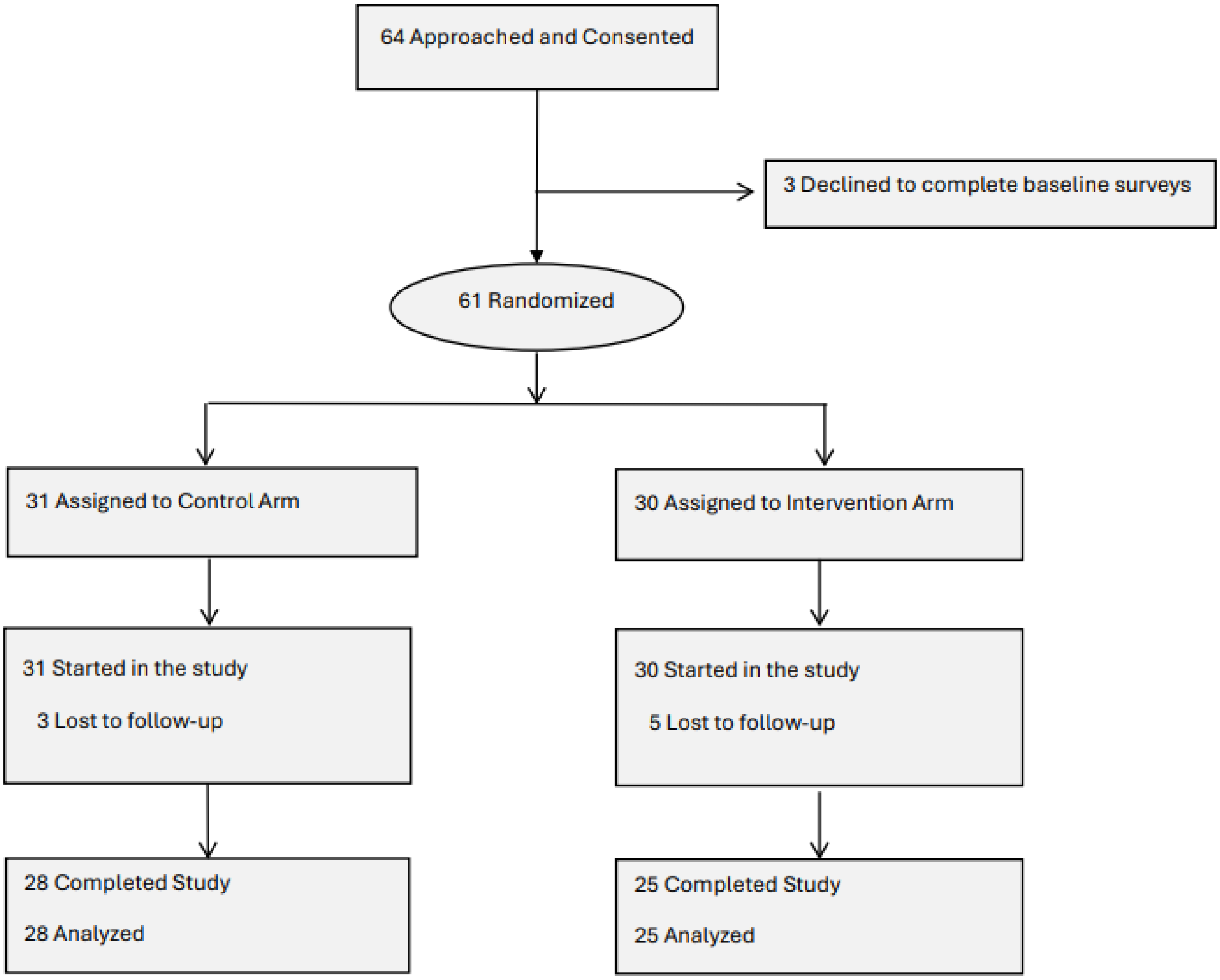

© 2025 Lee D et al. *JAMA Network Open*.
