# Supplementary material for: Gamification With a Support Partner and Postoperative Mobility in Older Adults Undergoing Radical Cystectomy: The MOVE UP Randomized Clinical Trial
Source: JAMA Netw Open. 2025 Jan 13;8(1):e2453037. doi: 10.1001/jamanetworkopen.2024.53037 (PMC11731219; doi:10.1001/jamanetworkopen.2024.53037)
Supplement: Supplement 3. — Data Sharing Statement [file jamanetwopen-e2453037-s003.pdf]

Data Sharing Statement

Lee. Effect of Gamification With a Support Partner to Increase Postoperative Mobility in Older Adults Undergoing Radical Cystectomy. *JAMA Netw Open*. Published January 13, 2025. doi:10.1001/jamanetworkopen.2024.53037

Data
Additional Information: Clincaltrials.gov NCT04314778
Data available: Yes
Data types: Deidentified participant data
How to access data: ryan.greysen@pennmedicine.upenn.edu
When available: With publication

Supporting Documents
Document types: None

Additional Information
Who can access the data: researchers whose proposed use of the data has been approved
Types of analyses: for a specified purpose
Mechanisms of data availability: with a signed data access agreement
